# PARP-1 and p53 Regulate the Increased Susceptibility to Oxidative Death of Lymphocytes from MCI and AD Patients

**DOI:** 10.3389/fnagi.2017.00310

**Published:** 2017-10-05

**Authors:** Felipe Salech, Daniela P. Ponce, Carol D. SanMartín, Nicole K. Rogers, Carlos Chacón, Mauricio Henríquez, Maria I. Behrens

**Affiliations:** ^1^Facultad de Medicina, Instituto de Ciencias Biomédicas, Universidad de Chile, Santiago, Chile; ^2^Centro de Investigación Clínica Avanzada (CICA), Hospital Clínico Universidad de Chile, Santiago, Chile; ^3^Departamento de Neurociencias, Facultad de Medicina, Universidad de Chile, Santiago, Chile; ^4^Departamento de Neurología y Neurocirugía, Hospital Clínico Universidad de Chile, Santiago, Chile; ^5^Clínica Alemana de Santiago, Santiago, Chile

**Keywords:** PARP-1, p53, oxidative cell death, lymphocytes, AD, MCI

## Abstract

Mild cognitive impairment (MCI) is a clinically detectable initial stage of cognitive deterioration with a high conversion rate to dementia. There is increasing evidence that some of the cerebral alterations present in Alzheimer type dementia can be found in peripheral tissues. We have previously shown that lymphocytes from Alzheimer’s disease (AD) patients have increased susceptibility to hydrogen peroxide (H_2_O_2_)-induced death that depends on dementia severity. We here investigated whether lymphocytes from MCI patients show increased vulnerability to death, and explored the involvement of Poly [ADP-ribose] polymerase (PARP-1) and p53 in the regulation of this process. Lymphocytes from 16 MCI and 10 AD patients, and 15 healthy controls (HCs) were submitted to increasing concentrations of H_2_O_2_ for 20 h. Cell death was determined by flow cytometry, in the presence or absence of PARP-1 inhibitors (3-aminobenzamide (3-ABA) or Nicotinamide (NAM)), or the p53 inhibitor (nutlin-3) or stabilizer (pifithrin-α). PARP-1 and p53 mRNA levels were determined by quantitative PCR (qPCR). Lymphocytes from MCI patients showed increased susceptibility to death, attaining intermediate values between AD and controls. PARP inhibitors -3-ABA and NAM- markedly protected from H_2_O_2_-induced death, making the difference between MCI and controls disappear, but not the difference between AD and controls. PARP-1 mRNA expression was increased in MCI lymphocytes. Modulation of p53 with Nutlin-3 or pifithrin-α did not modify the H_2_O_2_-induced death of lymphocytes from MCI or AD patients, but augmented the death in control lymphocytes attaining levels similar to MCI and AD. Accordingly, p53 mRNA expression was increased in AD and MCI lymphocytes compared to controls. In all, these results show that increased oxidative death is present in lymphocytes at the MCI stage. PARP-1 has a preponderant role, with complete death protection achieved with PARP inhibition in MCI lymphocytes, but not in AD, suggesting that PARP-1 might have a protective role. In addition, deregulations of the p53 pathway seem to contribute to the H_2_O_2_-induced death in MCI and AD lymphocytes, which show increased p53 expression. The results showing a prominent protective role of PARP inhibitors opens the door to study the use of these agents to prevent oxidative death in MCI patients.

## Introduction

Alzheimer’s disease (AD) is the most frequent cause of dementia, affecting an estimated 30 million people worldwide (Prince et al., 2016). Aging is the main risk factor to develop the disease: the annual incidence increases from 1% to 2% at the ages of 65 and doubles every 5 years thereafter (Querfurth and LaFerla, 2010). Considering the increase in population aging, it is expected that the number of patients with AD will increase progressively during the next years. Mild cognitive impairment (MCI)—a cognitive decline without functional deficit—especially the amnestic type, precedes the earliest manifestations of AD, defining a clinically detectable initial stage of the disease (Petersen, 2011). The possibility that interventions during the stage of MCI may change the clinical course of the disease has led to a significant increase in the study of this condition. The diagnosis of MCI and AD are based on clinical evaluation, neuropsychological evaluation and neuroimaging to support the diagnosis. However, there is still a necessity of a simple and reliable biomarker to diagnose the disease (Olsson et al., 2016).

It is increasingly recognized that AD is a systemic disorder that affects different peripheral tissues in addition to the pathology in the brain (Ray et al., 2007; Arosio et al., 2014; Khan and Alkon, 2015). We have reported that lymphocytes from AD patients show an increased susceptibility to hydrogen peroxide (H_2_O_2_)-induced cell death compared with lymphocytes from healthy controls (HCs; Behrens et al., 2012). Furthermore, we showed that this increased susceptibility to oxidative death was dependent on the severity of the dementia, being greater with more advanced dementia (Ponce et al., 2014). Whether this enhanced vulnerability is present in the initial stages of the disease, in MCI patients, is unknown. The mechanism by which lymphocytes of AD patients show enhanced susceptibility to H_2_O_2_-induced cell death is not clear. Using flow cytometry, electron microscopy and by measuring caspase activity, we previously reported that H_2_O_2_ exposition induces both necrotic and apoptotic death, the latter being independent of caspase activity (Behrens et al., 2011). H_2_O_2_ is known to generate reactive oxygen species (ROS) that produce DNA damage. This injury activates cell repair pathways such as p53 and poly [ADP-ribose]-polymerase-1 (PARP-1) that depending on the extent of damage can initiate DNA repair mechanisms or activate programmed cell death, inducing either apoptosis (p53), or a caspase-independent, PARP-1-dependent cell death (Lakin and Jackson, 1999; Jagtap and Szabó, 2005; Green and Kroemer, 2009). Experimental data suggest that both pathways might participate in the regulation of AD lymphocyte oxidative cell death (Uberti et al., 2008). In previous studies, we reported a marked protective role of 3-aminobenzamide (3-ABA)—a pharmacological inhibitor of PARP-1—in reducing H_2_O_2_-induced cell death in AD lymphocytes (Behrens et al., 2012; Ponce et al., 2014). Also, increased expression and levels of an unfolded form of p53 have been reported in AD lymphocytes (Uberti et al., 2008; Lanni et al., 2010; Bialopiotrowicz et al., 2012; Tan et al., 2012). The aim of this study was to determine whether the increased lymphocyte susceptibility to H_2_*O*_2_-induced death is present in MCI patients and to investigate the involvement of PARP-1 and p53 in the regulation of this process.

## Materials and Methods

### Patients

A total of 41 individuals: 16 MCI, 10 AD patients and 15 healthy donors were recruited. This study was carried out in accordance with the recommendations of the Ethics Committee of the Hospital Clínico de la Universidad de Chile with written informed consent from all subjects. All subjects gave written informed consent in accordance with the Declaration of Helsinki. The protocol was approved by the Ethics Committee of the Hospital Clínico de la Universidad de Chile. Caregivers of patients with severe dementia provided the consent. Three patients from the AD group were analyzed in a previous report (Behrens et al., 2012) but donated new blood samples. AD diagnosis was established following the guidelines of Alzheimer’s Association and the National Institute on Aging (McKhann et al., 2011). Dementia severity was rated with the clinical dementia rating clinical dementia rating (CDR; Morris, 1997) and with the Montreal Cognitive Assessment (MoCA) test validated in Spanish in our country (Delgado et al., 2017). The maximum score for the MoCA is 30, with lower scores associated with greater cognitive deterioration. HCs were submitted to the same neurological and neuropsychological evaluations. Table 1 shows the demographic data of study participants.

**Table 1:** Demographic table of the study participants.

	Healthy controls *n* = 15	MCI *n* = 16	AD *n* = 10
Age, mean ± SE (range)	74.1 ± 1.2 (69–85)	77.0 ± 1.8 (60–89)	79.0 ± 1.8 (68–84)
Female sex, (%)	12 (80)	12 (75)	9 (90)
Education	11.6 ± 0.8	9.1 ± 1.1	10.2 ± 1.5
CDR	0	0.5	2.5 ± 0.2
MoCA test score mean ± SE	28.1 ± 0.8	20.2 ± 1.1	5.4 ± 1.6
Diabetes or insulin resistance, (%)	5 (33)	2 (13)	2 (20)
Hypertension, (%)	7 (47)	7 (44)	5 (50)
Ever smoked tobacco, (%)	4 (27)	2 (13)	4 (40)

*Abbreviations: MCI, Mild Cognitive Impairment; AD, Alzheimer’s disease; CDR, Clinical Dementia Rating; MoCA, Montreal Cognitive Assessment*.

### Materials and Equipment

Ficoll-Hypaque™ PLUS was from GE Healthcare (Little Chalfont, RU), H_2_O_2_ was from Merck (Darmstadt, Germany), 3-ABA, Nicotinamide (NAM), Pifithrin-α and Nutlin-3 were from Sigma-Aldrich (Oakville, ON, Canada), Trizol was from Life Technology (Carlsbad, CA, USA), TURBO DNA- free™ Kit was from Invitrogen (Waltham, MA, USA), High Capacity cDNA Reverse Transcription Kit was from Thermo Fisher Scientific (Carlsbad, CA, USA), Brilliant III SYBER-GREEN Master Mix and MX3000P were from Agilent Technologies (La Jolla, CA, USA). Flow cytometry FACScan was from Becton Dickinson (Franklin Lakes, NJ, USA).

### Peripheral Blood Lymphocytes (PBL)

Peripheral blood lymphocytes (PBL) were obtained by venipuncture (15 ml) and extracted by Ficoll-Hypaque density centrifugation. Lymphocytes were exposed to increasing concentrations of H_2_O_2_ for 20 h (Behrens et al., 2011, 2012; Ponce et al., 2014). The effect of PARP-1 inhibition on H_2_O_2_-induced death was evaluated by the addition of 3-ABA (5 mM) or NAM (5 mM,), and the effect of p53 modulation by the addition of the p53 inhibitor Pifithrin-α (20 μM), or the p53 stabilizer Nutlin-3 (10 μM), added 30 min before H_2_O_2_ exposure. Samples containing roughly 1 × 10^6^ cells were analyzed by flow cytometry following propidium iodide (PI) staining, in which viable (PI-negative), apoptotic (PI-positive, hypodiploid) and necrotic (PI-positive diploid) cells were distinguished (Behrens et al., 2011, 2012; Ponce et al., 2014).

### RNA Isolation and PCR Analysis

Total RNA was isolated using Trizol reagent. To remove any contaminating genomic DNA, a DNAase digestion step with TURBO DNA- free™ Kit was included. RNA purity was assessed by the 260/280-absorbance ratio. cDNA was synthesized from total RNA (2 μg) using the High Capacity cDNA Reverse Transcription Kit. Real-time quantitative PCR (qPCR) was performed in an amplification system MX3000P, using the DNA binding dye SYBR green (Brilliant III SYBER-GREEN Master Mix). Amplification was performed using the following primers: PARP-1: 5′-TTGAAAAAGCCCTAAAGGCTCA-3′, 5′-CTACTCGGTCCAAGATCGCC-3′. P53: 5′-AGCTTTGAGGTGCGTGTTTG-3′, TCAGCTCTCGGAACATCTCG-3′. 18S: 5′-GATATGCTCATGTGGTGTTG-3′, AATCTTCAGTCGCTCCCA-3′. Levels of PARP-1 and p53 mRNA were normalized with respect to levels of 18S mRNA. Quantification was performed using the technique ΔΔCt (Pfaffl, 2001). Dissociation curves were analyzed to verify purity of products. All samples were run in triplicate.

### Statistical Analysis

Differences between the three experimental groups at each dose in lymphocyte survival, apoptosis and necrosis, adjusted for age and sex, were analyzed using SPSS general linear model. Data from qPCR were analyzed using analysis of variance (ANOVA) with Bonferroni correction. Results were expressed as means ± standard error of the mean (SEM). Differences *p* ≤ 0.05 were considered statistically significant.

## Results

### Increased Cell Death Susceptibility in Lymphocytes from MCI Patients

**Figure 1:**
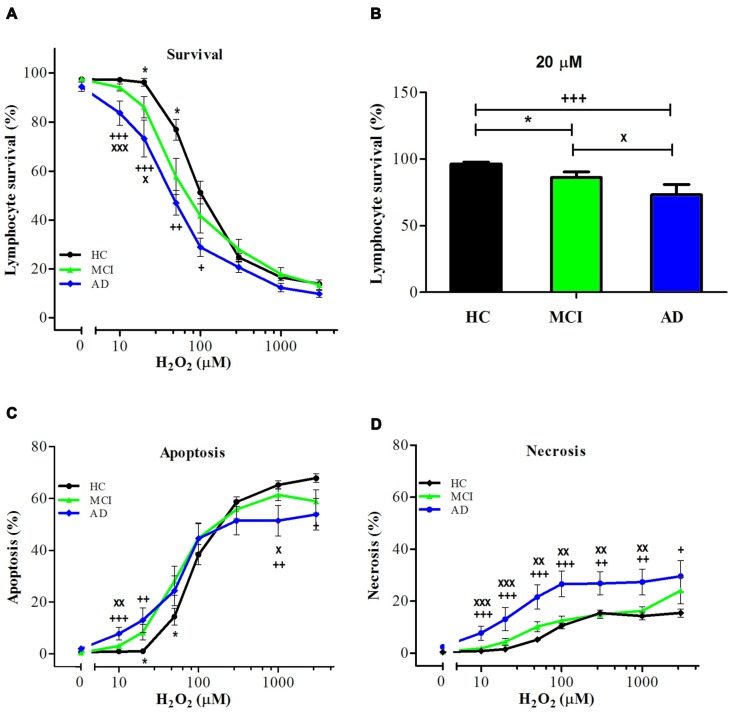
Hydrogen peroxide (H_2_O_2_)-induced death of lymphocytes from mild cognitive impairment (MCI) and Alzheimer’s disease (AD) patients and healthy controls (HCs). Lymphocytes from 16 MCI patients (green symbols), 10 AD patients (blue symbols) and 15 (HC; black symbols) were exposed to H_2_O_2_ for 20 h and death was determined by flow cytometry with propidium iodide (PI) staining. **(A)** Lymphocyte survival curve at increasing concentrations of H_2_O_2_; **(B)** survival values at 20 μM H_2_O_2_; **(C,D)** apoptosis and necrosis curves from experiments in **(A)**, respectively (%, means ± SE). *MCI vs. HC; ^+^AD vs. HC; ^x^AD vs. MCI clinical dementia rating (CDR) 0.5. 1 symbol: *p* < 0.05; 2 symbols: *p* < 0.005; 3 symbols: *p* < 0.0001 for all figures.

Upon exposure to H_2_O_2_, lymphocytes from MCI patients showed increased susceptibility to death compared with control lymphocytes (Figure 1A). The H_2_O_2_ dose-response curves of lymphocyte viability (concentrations ranging from 10 μM to 3 mM) were shifted to the left (enhanced sensitivity) in MCI lymphocytes compared to HC, attaining intermediate values between controls and AD patients (Figure 1A). Upon treatment with 20 μM H_2_O_2_, survival values were 73.2 ± 7.6%, 86.1 ± 6.2% and 96.3 ± 6.3% for AD, MCI and HC lymphocytes, respectively (Figure 1B). When examining the type of death induced by H_2_O_2_, MCI lymphocytes showed increased apoptosis compared with control lymphocytes, without changes in necrosis (Figures 1C,D). Instead AD patients showed increased apoptosis and also a significant increase in necrosis (Figures 1C,D).

### PARP-1 in the Regulation of Oxidative Cell Death of Lymphocytes from MCI and AD Patients

**Figure 2:**
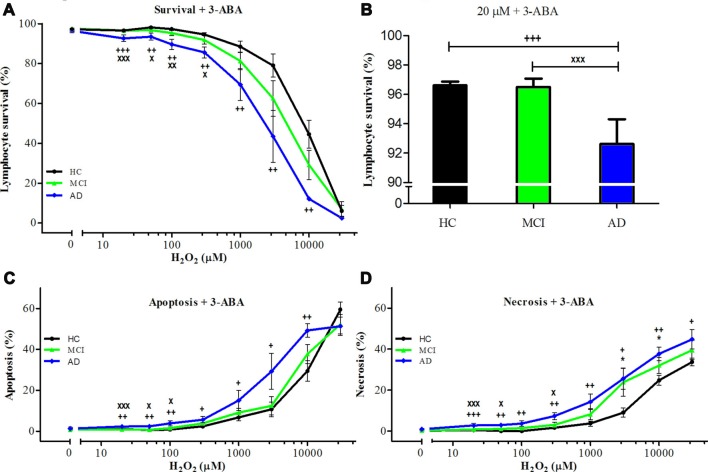
Effect of poly [ADP-ribose] polymerase (PARP-1) inhibition with 3-aminobenzamide (3-ABA) on H_2_O_2_-induced death of lymphocytes. Lymphocytes from 16 MCI patients (green symbols), 10 AD patients (blue symbols) and 15 (HCs; black symbols) were pre-incubated with 5 mM 3-ABA for 30 min before the exposure to H_2_O_2_ for 20 h. **(A)** survival curves (%, means ± SE); **(B)** survival values at 20 μM H_2_O_2_ (%, mean ± SE); **(C,D)** apoptosis and necrosis curves, respectively from experiments in (**A**; % means ± SE). Symbols as in Figure 1.

**Figure 3:**
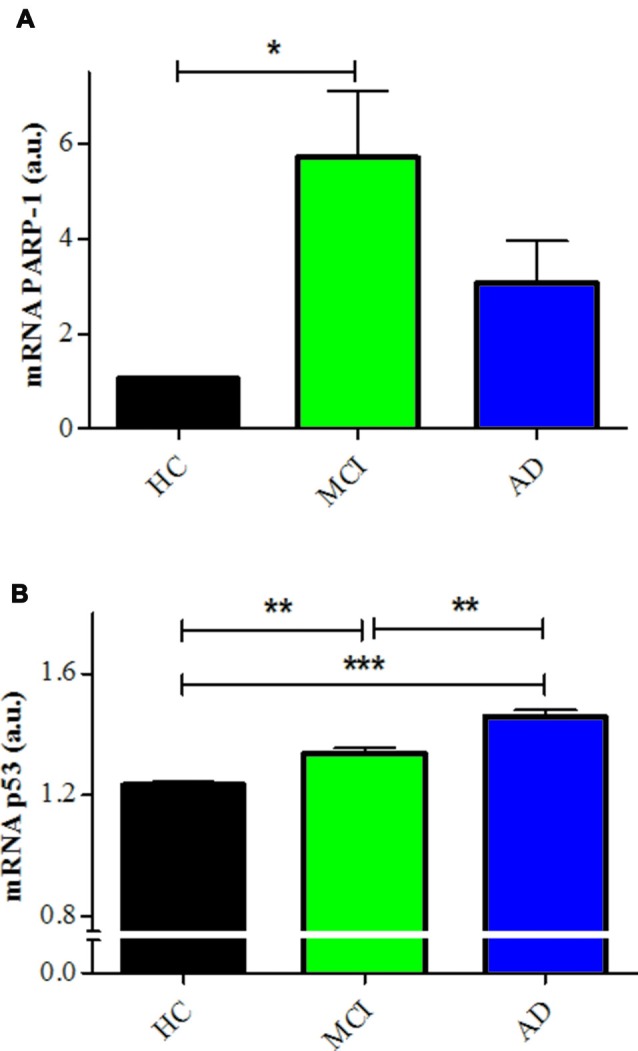
PARP-1 and p53 mRNA expression in lymphocytes.** (A)** mRNA levels of PARP-1 measured by quantitative PCR (qPCR; means ± SE). HCs (*n* = 5); MCI (*n* = 5); AD (*n* = 3). **(B)** mRNA levels of p53 determined by qPCR (means ± SE). *n* = 5 for HC, MCI and AD patients. *p < 0.05; **p < 0.005; ***p < 0.0001.

The inhibition of PARP-1 with 3-ABA, produced a marked reduction in the H_2_O_2_-induced cell death in all groups, inducing the disappearance of the difference between MCI and control lymphocytes (Figures 2A,B). However, AD lymphocytes maintained a significantly increased susceptibility to death inhibition compared with control lymphocytes (Figures 2A,B), as was reported previously (Ponce et al., 2014). An increase in 3-ABA concentration did not modify these results suggesting that the difference was not due to insufficient PARP-1 inhibition (data not shown). The examination of the type of death rescued by 3-ABA showed that, in HC lymphocytes, the protection granted by PARP-1 inhibition was achieved by a decrease in apoptosis, without major changes in necrosis (Supplementary Figure S1). Instead, under 3-ABA treatment MCI lymphocytes showed similar levels of apoptosis compared with control lymphocytes, but increased levels of necrosis under high H_2_O_2_ concentrations, whereas AD lymphocytes showed significant increase in both apoptosis and necrosis (Figures 2C,D). Consistently, the use of NAM, another recognized inhibitor of PARP-1 activity, provoked very similar effects compared to 3-ABA (Supplementary Figures S2A–D). Therefore, the death of lymphocytes caused by H_2_O_2_ exposure was preponderantly dependent on the PARP-1 pathway in the three groups of patients, however in MCI lymphocytes the protection by PARP-1 inhibition was the same as in controls, whereas in AD the protection was incomplete. Considering the important functional effect of pharmacological PARP-1 inhibition, we explored whether there were changes in the basal mRNA expression levels of PARP-1 in MCI and AD lymphocytes. Using qPCR we found that MCI lymphocytes had significantly higher expression levels of PARP-1 mRNA (Figure 3A). Lymphocytes from AD patients had levels not significantly different from controls (Figure 3A). This result might indicate a protective role for PARP-1 at initial stages of the disease.

### p53 in the Regulation of Oxidative Cell Death of Lymphocytes from MCI and AD Patients

**Figure 4:**
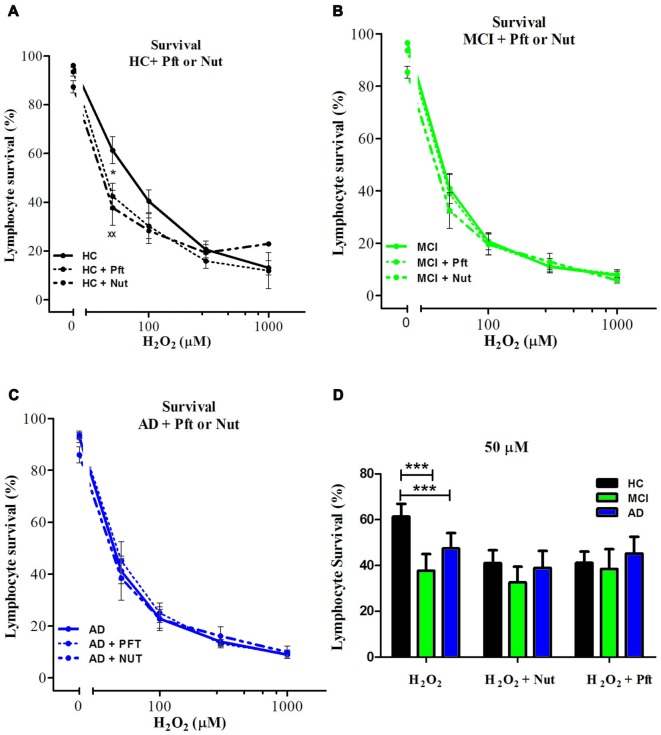
Effect of p53 modulation on H_2_O_2_-induced death of lymphocytes. Lymphocytes from six HCs **(A)**, eight MCI patients **(B)** and five AD patients **(C)** were exposed to H_2_O_2_ for 20 h in the absence (continuous line) or the presence of 20 μM Pifithrin-α (Pft), a p53 inhibitor (short interrupted lines), or 10 μM Nutlin 3a (Nut), a p53 stabilizer, (long interrupted lines), applied 30 min before H_2_O_2_ incubation (means ± SE); **(D)** Lymphocyte survival values measured at 50 μM H_2_O_2_ with Pft or Nut (means ± SE). Symbols: *H_2_O_2_ vs. H_2_O_2_ + Pft; ^x^H_2_O_2_ + Pft vs. H_2_O_2_ + Nut. 1 symbol: *p* < 0.05; 2 symbols: *p* < 0.005; 3 symbols: *p* < 0.0001 for all figures.

The incomplete protective effect exerted by 3-ABA in AD lymphocytes and the lack of difference in the expression of PARP-1 suggest that there might be other mechanisms involved in the increased susceptibility to death of AD lymphocyte. Since oxidative damage is known to activate p53, we studied whether this pathway has a role in the different pattern of cell death observed between AD and control lymphocytes. Pharmacological modulation of p53 provoked changes in the susceptibility to death of control lymphocytes, but not of MCI or AD lymphocytes (Figure 4A). Stabilizing p53 with nutlin-3 induced an increase in cell death of HC lymphocytes that reached the levels observed in MCI and AD (Figure 4D). Unexpectedly, the addition of the p53 inhibitor pifithrin-α also induced an increase in cell death of HC lymphocytes that was significant at 50 μM (Figures 4A,D). On the other hand, neither the addition of nutlin-3 or pifithrin-α induced changes in the H_2_O_2_-induced death of MCI or AD lymphocytes (Figures 4B–D), suggesting that p53 might be already activated in lymphocytes from MCI and AD patients. Consistent with these results, the expression levels of p53 mRNA were increased both in MCI and AD lymphocytes compared to controls (Figure 3B), with a significantly higher expression in AD compared with MCI lymphocytes (Figure 3B).

When analyzing the type of death induced by p53 stabilization in HC lymphocytes, the increased death was due to increased necrosis, without changes in apoptosis and a similar effect was seen upon p53 inhibition (Supplementary Figure S3, left panels), suggesting that changes in necrosis are preferred whenever there is a modulation of p53. Although inhibition or stabilization of p53 had no effect on overall survival of MCI and AD lymphocytes, p53 inhibition caused a decrease in apoptosis in MCI lymphocytes that was accompanied by a proportional increase in necrosis, and a similar tendency was observed in AD lymphocytes (Supplementary Figure S3, middle and right panels). Instead, p53 stabilization had no effect either in apoptosis or necrosis both in MCI and AD lymphocytes (Supplementary Figure S3, middle and right panels).

## Discussion

We here report that the increased susceptibility to cell death in AD lymphocytes is already detectable at the MCI stage of the disease. Both PARP-1 and p53 pathways play a role in the process of oxidative cell death of lymphocytes, with a preeminent role of PARP-1, however, a deregulation of the p53 pathway also seems to play a role in the increased death observed in AD and MCI lymphocytes.

The increased susceptibility to cell death in MCI lymphocytes is lower than in AD lymphocytes, which is in agreement with our previous reports showing that the susceptibility to cell death worsens with the progression of the illness (Ponce et al., 2014). Therefore, this phenomenon is present at initial stages of the disease which may be useful for early diagnosis.

In addition, in MCI the type of death is predominantly apoptosis, whereas in AD patients there are increased levels of both, apoptosis and necrosis. The group of AD patients in this study were more severely affected than those in our previous work, which showed only increases in apoptosis (Ponce et al., 2014). Therefore, it is possible that with advancement of the disease in addition to an increase in susceptibility to oxidative death, there is also a change in the type of death of lymphocytes; initially with an increase in apoptosis and later on with increases in both apoptosis and necrosis.

Cells can die by different mechanisms. The most well-known is apoptosis, a form of programmed cell death associated with the liberation of cytochrome c by the mitochondria and activation of caspases, leading to DNA fragmentation and cell death (Hengartner, 2000; Taylor et al., 2008). In opposition, necrosis, thought of as an unregulated type of cell death occurring by rupture of the cell membrane after swelling due to imbalance of electrolytes and a disorganized liberation of the cell contents, is now recognized as a regulated form of cell death too (Henriquez et al., 2008; Conrad et al., 2016). In fact, it is now recognized that unregulated necrosis only occurs in traumatic injuries, whereas in other physiopathological conditions several other forms of regulated necrosis exist (Linkermann et al., 2014), such as necroptosis, parthanatos, mitochondrial permeability transition (MPT)-mediated regulated necrosis (MPT-RN), ferroptosis and Netosis. It is also recognized that cells can die by different mechanisms that can coexist and modulate each other; in fact, the activation of one type of death can modulate the development of other types of death, i.e., caspases are known to cleave and inhibit PARP-1 (D’Amours et al., 2001) and caspase eight has been shown to inhibit necroptosis (Oberst et al., 2011). Parthanatos is a recently described form of caspase-independent cell death that shares characteristics of apoptosis and necrosis and is dependent on the activation of PARP-1 (Fatokun et al., 2014; Dawson and Dawson, 2017). PARP-1 is a nuclear enzyme that participates in DNA repair by rapidly synthesizing PAR molecules that parylate proteins within a few minutes, forming a scaffold that facilitates DNA repair by other enzymes. The activity of PARP-1 consumes NAD^+^ and therefore, in conditions of intense DNA damage, such as oxidation by H_2_O_2_, the NAD^+^ and ATP contents of the cell are depleted, leading to cell death, which is no longer by apoptosis—since apoptosis requires ATP—but by parthanatos. In parthanatos, PAR molecules are released into the cytoplasm stimulating the liberation of the apoptosis-inducing factor (AIF) from mitochondria that translocates to the nucleus, producing chromatin condensation and large-scale DNA fragmentation and cell death (Wang et al., 2011). Interestingly, this type of death is observed in different cells of the organism and in neurons (Lee et al., 2013), and might be a form of cell death present in neurodegenerative disorders. There is evidence of a role of parthanatos in parkinsonism. It was demonstrated that the toxic effect of 1-Methyl-4-phenyl-1,2,3,6-tetrahydropyridine (MPTP), a toxin that induces parkinson-like symptoms in humans (Langston et al., 1983), is reduced in mutated cells lacking the PARP gene (Mandir et al., 1999). The fact that the death observed in lymphocytes in our study was so significantly protected by PARP-1 inhibition lead us to suggest that it most probably corresponds to Parthanatos. In addition, in our previous report we demonstrated that the death of lymphocytes induced by H_2_O_2_ exposure was not associated with changes in caspase activity, nor affected by the caspase inhibitor, zVAD (Behrens et al., 2011). It would be interesting to study other downstream metabolites, such as AIF translocation from mitochondria to nucleus. However, there are reports indicating that parthanatos can occur without changes in AIF (Jang et al., 2017), and the increase in PARP activity remains the hallmark of this type of death.

Usually parthanatos is considered a necrosis type of death (Linkermann et al., 2014), however, it shares characteristics of both apoptosis and necrosis (Zhang et al., 2015). In fact, electron microscopy of lymphocyte H_2_O_2_-induced death displayed characteristics of both apoptosis, such as chromatin condensation and blebbing, and also of necrosis, with cellular and organelle swelling (Behrens et al., 2011). We here found that the death caused by H_2_O_2_ of HC lymphocytes, measured by flow cytometry, was accounted mostly by apoptosis and the protective effect of PARP-1 inhibition was mainly induced by reducing apoptosis, therefore, suggesting that oxidative stress of lymphocytes induces a PARP-1 dependent-apoptosis type of death (Behrens et al., 2011, 2012; Ponce et al., 2014).

Besides PARP-1 preponderant involvement in the death of lymphocytes, there seems to be a participation of p53 in the regulation of oxidative death of lymphocytes. p53 is a transcription factor known as the guardian of the genome by its role in sensing DNA damage and inducing DNA repair, or if that is not possible, leading to either cell cycle arrest, apoptosis, or senescence (Lakin and Jackson, 1999; Green and Kroemer, 2009). PARP-1 shares this role as guardian of the cell. However, the interplay between p53 and PARP inducing cell death is not clear (Montero et al., 2013; Elkholi and Chipuk, 2014; Ying and Padanilam, 2016). We here show evidence that lymphocyte death induced by oxidative damage seems to involve both, PARP-1-dependent also p53-dependent type of deaths. We observed that p53 stabilization with nutlin-3, and also p53 inhibition with pifithrin-α, caused increased death due to greater necrosis that reached the levels attained by MCI and AD lymphocytes. The fact that both inhibition and stabilization of p53 induced a decrease in survival in HC lymphocytes suggests that the role of p53 in HC might be to maintain the equilibrium of cell survival regulation, with all deviations from this level inducing cells to fall into necrosis. Differing from the effect in HC, in MCI and AD lymphocytes stabilization or inhibition of p53 had no effect on lymphocyte survival; suggesting that the p53 pathway might be already activated in AD and MCI. A disruption of the p53 pathway is in accordance with the increased levels of p53 mRNA observed in MCI lymphocytes that were even higher in AD. Higher levels of p53 mRNA expression in lymphocytes from AD and MCI patients are consistent with other studies (Uberti et al., 2008; Lanni et al., 2010; Zhou and Jia, 2010; Buizza et al., 2012; Tan et al., 2012), however, it would be interesting to evaluate the different conformations of p53 that have been previously described (Uberti et al., 2008; Lanni et al., 2010; Buizza et al., 2012). Also, a lack of p53 regulation is in accordance with the results of Zhou and Jia (2010) reporting a p53-mediated G(1)/S checkpoint dysfunction in lymphocytes from AD patients. MCI lymphocytes also showed increased PARP-1 mRNA levels that were not significantly augmented in AD lymphocytes. This might reflect an initial protective mechanism to prevent oxidative death.

The survival results in our study were adjusted for age and gender, as described in “Materials and Methods” Section. The age range in the three groups of patients was similar, however the AD and MCI groups were older than the controls. In our previous work in control subjects we demonstrated that overall lymphocyte survival was independent of the age of the subjects, although there was an increase in the apoptosis/necrosis ratio. Whether lymphocyte death is independent of age in AD subjects is not known, however the number of participants in this study is insufficient to analyze it. The majority of participants in our study were women, as is usual in these studies. There are reports in rat hippocampal neurons indicating that male neurons had greater susceptibility to oxidative and excitotoxic death, and more AIF-mediated death compared with female neurons, in which cytochrome c-mediated death predominated (Du et al., 2004), but there are no reports in humans. However, as with age the small number of male participants in our study precludes this analysis in this study.

In all, these results suggest that both PARP-1 and p53 are involved in the increased susceptibility to death observed in MCI and AD patients, with a deregulation of the p53 pathways that probably increases as the disease progresses. The involvement of other cell death mechanisms, such as autophagy or necroptosis, cannot be ruled out with the present experiments, since cells are capable of activating multiple types of death. However, the presence of other types of death seems not to be the case, given the fact that PARP-1 inhibition prevented almost 90% of death (at 1 mM H_2_O_2_).

In MCI lymphocytes PARP-1 inhibition was capable of an almost complete prevention of the increased oxidative death, by decreasing apoptosis, whereas in patients with AD, where the death also depends on necrosis, the effect was only partial. Therefore, these results suggest that progression of the disease might progressively involve apoptosis and then also necrosis. Following the same idea, the results indicate that PARP-1 inhibition might be useful at initial stages of the disease, consistent with the existence of a therapeutic window in which it would be possible to intervene on the phenomenon of cell death in early stages of neurodegeneration. PARP inhibitors, such as NAM, are simple interventions that might be explored as a treatment to prevent oxidative cell death in MCI patients. If lymphocyte death is a reflection of overall cell death in the organism, it might also prevent neuronal death.

Finally, the good correlation between the severity of the disease and the lymphocyte cell death susceptibility showed by our results also add to the increasing evidence that peripheral tissues show changes in neurodegenerative disorders, which represent a much feasible tissue to investigate the mechanisms of disease.

## Author Contributions

FS, DPP and MIB: substantial contributions to the conception and design of the work; acquisition, analysis and interpretation of data; drafting and revising the work for important intellectual content; final approval of the version to be published. CDS: substantial contribution to design of the work; acquisition, analysis and interpretation of data; drafting and revising the work; final approval of the version to be published. NKR and CC: acquisition and analysis of data for the work; final approval of the version to be published. MH: substantial contributions to the design of the work, analysis and interpretation of data; final approval of the version to be published. All authors agree to be accountable for all aspects the work.

## Conflict of Interest Statement

The authors declare that the research was conducted in the absence of any commercial or financial relationships that could be construed as a potential conflict of interest.
