# Supplementary material for: PARP-1 and p53 Regulate the Increased Susceptibility to Oxidative Death of Lymphocytes from MCI and AD Patients
Source: Front Aging Neurosci. 2017 Oct 5;9:310. doi: 10.3389/fnagi.2017.00310 (PMC5633596; doi:10.3389/fnagi.2017.00310)
Supplement: Supplementary file 1 [file Image_1.pdf]

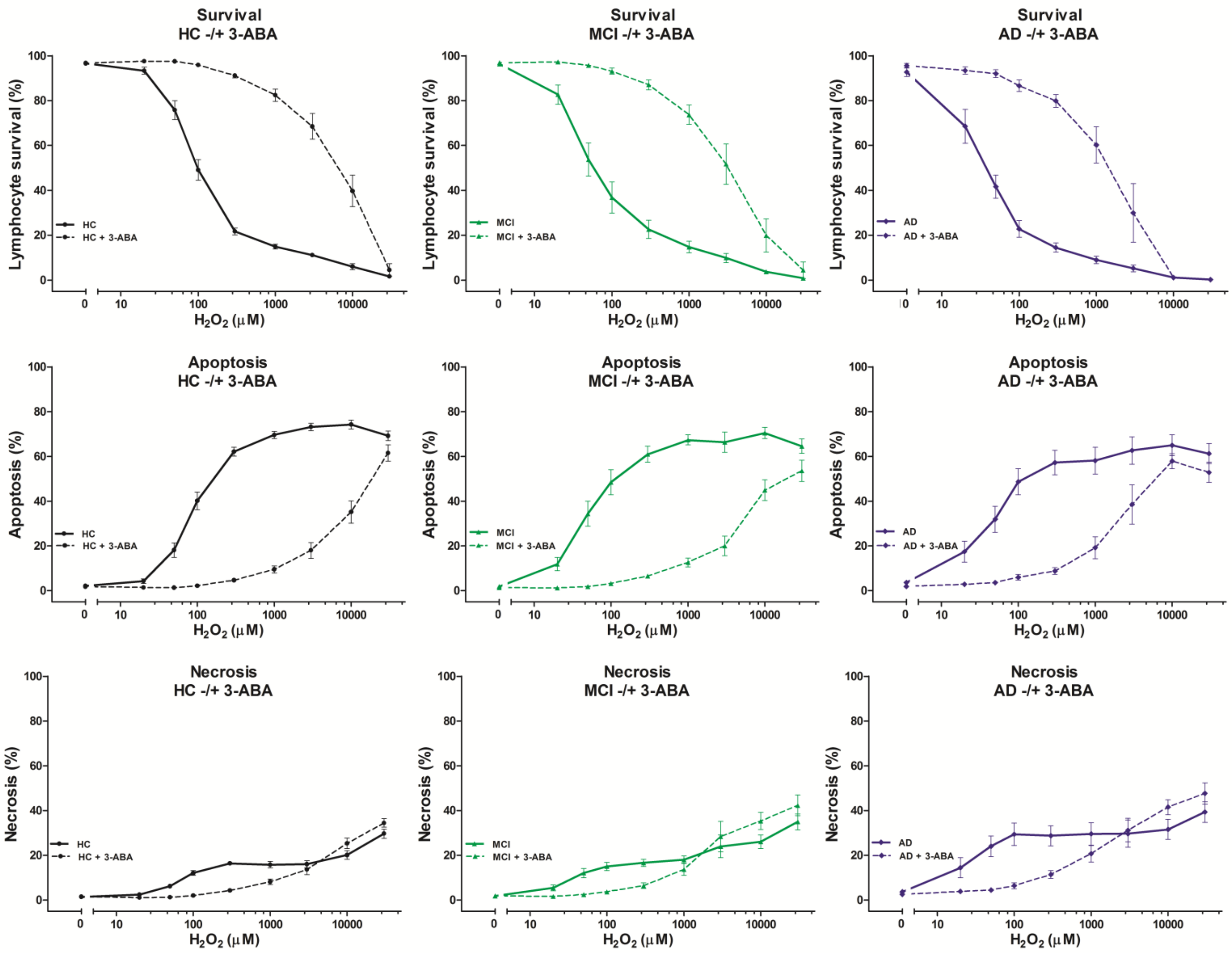

Supplementary Figure 1. Effect of PARP inhibition with 3-ABA on survival, apoptosis and necrosis of the H_2O_2-induced death of lymphocytes from HC, MCI and AD patients. Survival (upper panels), apoptosis (middle panels) and necrosis (lower panels) curves of the H_2O_2-induced death of lymphocytes from 15 healthy controls (HC; black symbols), 16 Mild Cognitive Impairment patients (MCI; green symbols), and 10 Alzheimer's disease patients (AD; blue symbols) were exposed to H_2O_2 for 20 hours in the absence (continuous line) or presence of 5 mM 3-ABA (interrupted lines), applied 30 min before H_2O_2 incubation (%, means ± SE*).*

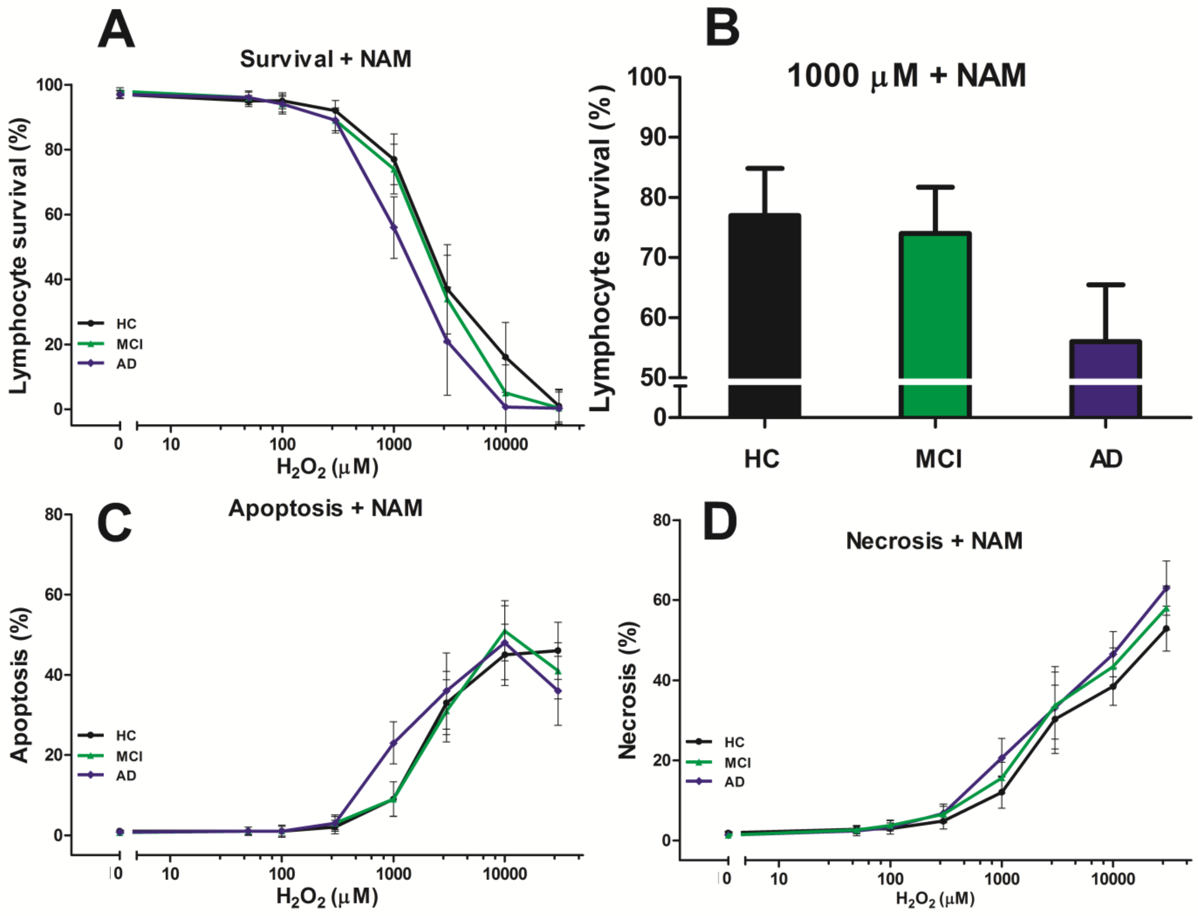

Supplementary Figure 2. Effect PARP-1 inhibition with Nicotinamide (NAM) on H_2O_2-induced death of lymphocytes. Lymphocytes from 8 Mild Cognitive Impairment patients (MCI; green symbols), 6 Alzheimer's disease patients (AD; blue symbols), and 5 healthy controls (HC; black symbols) were pre-incubated with 5 mM NAM for 30 minutes and then exposed to H_2O_2 for 20 hours . (**A**) Survival curves (means ± SE), (**B**) Survival at 1000 μM H_2O_2 (%, mean ± SE), (C) and (D) apoptosis and necrosis curves from experiments in A, respectively (%, means ± SE*).*

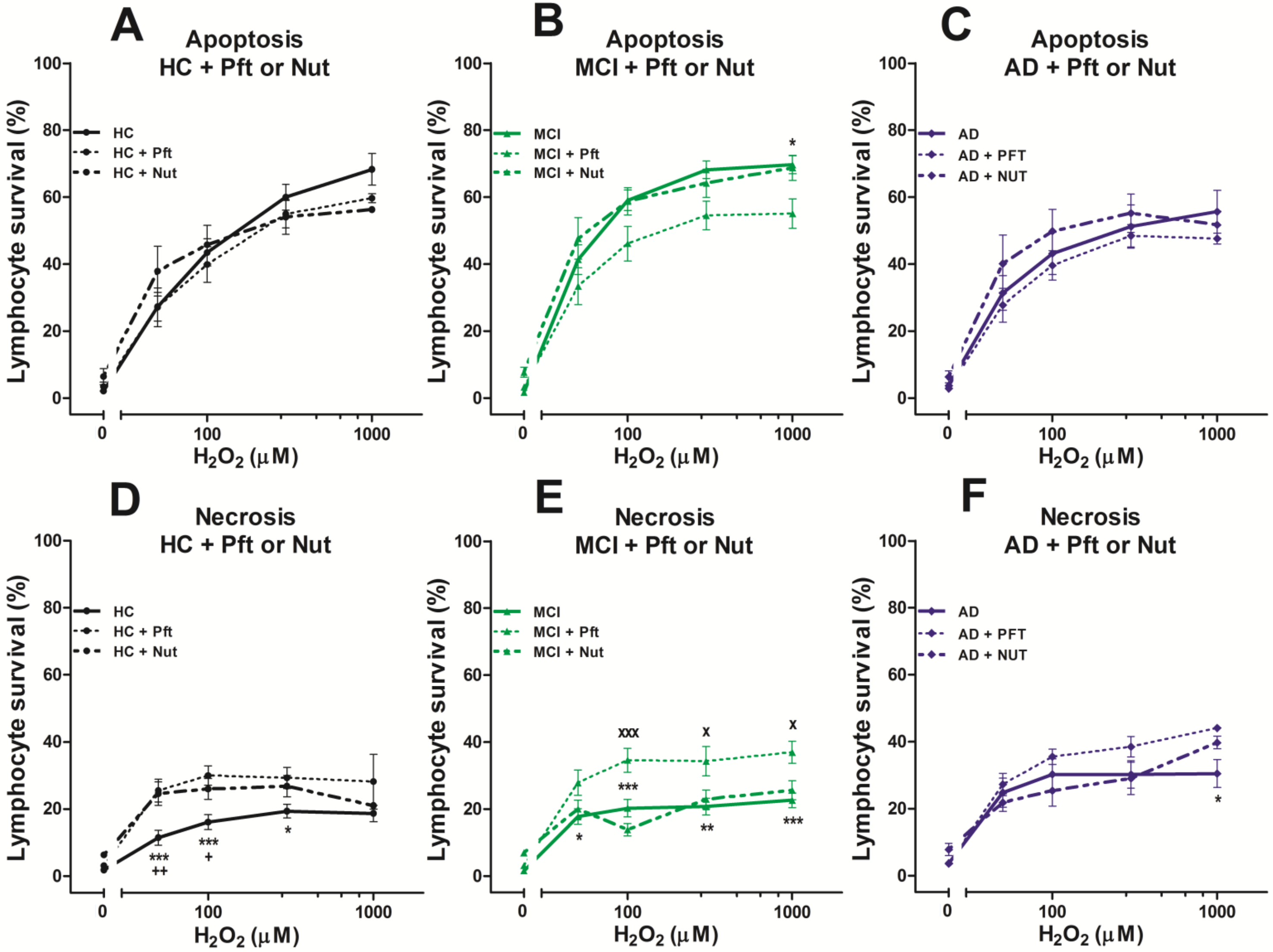

Supplementary Figure 3. Effect of p53 modulation on H_2O_2-induced apoptosis and necrosis of lymphocytes. Apoptosis (upper panels) and necrosis (lower panels) curves from the experiments in Fig 4 of the main document. Lymphocytes from 6 healthy controls (HC) (A), 8 Mild Cognitive Impairment patients (MCI) (B), and 5 Alzheimer's disease patients (AD) (C) were exposed to H_2O_2 for 20 hours in the absence (continuous line) or presence of the p53 inhibitor, Pifithrin-α (Pft) 20 μM (short interrupted lines), or the p53 stabilizer, Nutlin 3a (Nut) 10 μM (long interrupted lines) applied 30 min before H_2O_2 incubation (means ± SE*).* (D) Lymphocyte survival values measured at 50 μM H_2O_2 with Pft or Nut. (means ± SE*).* Symbols: *= H_2O_2 vs H_2O_2 + Pft; $^{+}$= H_2O_2 vs H_2O_2 + Nut; ^{x}= H_2O_2 + Pft vs H_2O_2 + Nut .1 symbol: $p<0.05$; 2 symbols: $p<0.005$; 3 symbols: $p<0.0001$ for all figures.
